# Ultrafast, All Optically Reconfigurable, Nonlinear Nanoantenna

**DOI:** 10.1021/acsnano.1c03386

**Published:** 2021-07-07

**Authors:** Eva Arianna Aurelia Pogna, Michele Celebrano, Andrea Mazzanti, Lavinia Ghirardini, Luca Carletti, Giuseppe Marino, Andrea Schirato, Daniele Viola, Paolo Laporta, Costantino De Angelis, Giuseppe Leo, Giulio Cerullo, Marco Finazzi, Giuseppe Della Valle

**Affiliations:** † Dipartimento di Fisica, Politecnico di Milano, Piazza Leonardo da Vinci, 32, I-20133 Milano, Italy; ‡ NEST, CNR-Istituto Nanoscienze and Scuola Normale Superiore, Piazza San Silvestro 12, 56127 Pisa, Italy; § Dipartimento di Ingegneria dell’Informazione, Università di Brescia, Via Branze 38, I-25123 Brescia, Italy; ∥ Matériaux et Phénomènes Quantiques, Université de Paris & CNRS, F-75013 Paris, France; ⊥ Istituto Italiano di Tecnologia, Via Morego 30, I-16163 Genova, Italy; # Istituto di Fotonica e Nanotecnologie, Consiglio Nazionale delle Ricerche, Piazza Leonardo da Vinci, 32, I-20133 Milano, Italy

**Keywords:** all-dielectric nanoantennas, second-harmonic generation, metasurfaces, ultrafast photonics, all-optical modulation

## Abstract

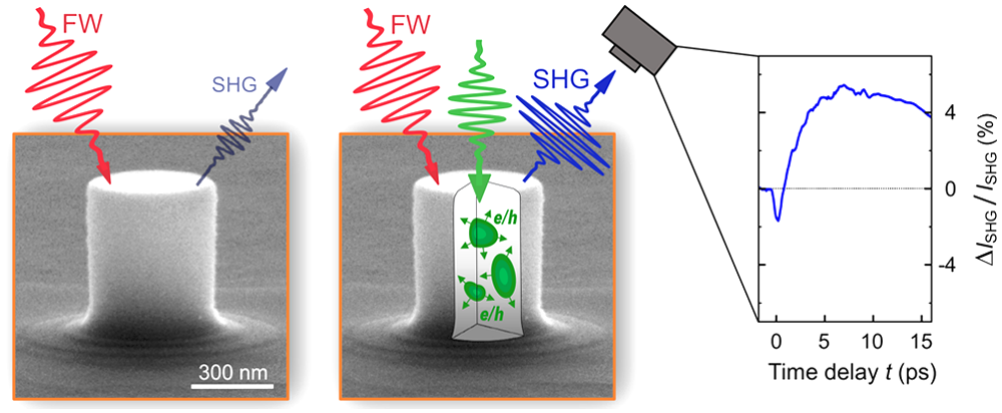

The enhancement of nonlinear optical effects *via* nanoscale engineering is a hot topic of research. Optical nanoantennas increase light–matter interaction and provide, simultaneously, a high throughput of the generated harmonics in the scattered light. However, nanoscale nonlinear optics has dealt so far with static or quasi-static configurations, whereas advanced applications would strongly benefit from high-speed reconfigurable nonlinear nanophotonic devices. Here we propose and experimentally demonstrate ultrafast all-optical modulation of the second harmonic (SH) from a single nanoantenna. Our design is based on a subwavelength AlGaAs nanopillar driven by a control femtosecond light pulse in the visible range. The control pulse photoinjects free carriers in the nanostructure, which in turn induce dramatic permittivity changes at the band edge of the semiconductor. This results in an efficient modulation of the SH signal generated at 775 nm by a second femtosecond pulse at the 1.55 μm telecommunications (telecom) wavelength. Our results can lead to the development of ultrafast, all optically reconfigurable, nonlinear nanophotonic devices for a broad class of telecom and sensing applications.

The capability to tailor the size and shape of high-index nanostructures has disclosed the opportunity to control light–matter interaction at the subwavelength scale, leading to the advent of nonlinear nanophotonics.^1−7^ For example, nanoscale engineering can lift phase-matching constraints typical of the bulk and turn centrosymmetric materials, such as gold, into efficient second-harmonic generation (SHG) media.^8^ Similarly, the optical Kerr effect in centrosymmetric nonlinear materials, such as silicon, has been enhanced by several orders of magnitude *via* nanoscale patterning.^9,10^ These advances were made possible by the capability of high-index nanostructures to simultaneously achieve intense local field enhancement and resonant light scattering, thus behaving as optical nanoantennas.^11−14^ Research in the field has been so far developed along two distinct directions characterized by different aims:^4^ (i) the enhancement of coherent harmonic generation for ultracompact nonlinear light sources;^6,7,15−19^ (ii) the engineering of giant delayed nonlinearities, induced by photogenerated carriers, for ultrafast light-controlling-light devices.^20−23^ Despite the huge efforts pursued on both topics, coherent nonlinear functionalities (such as second-/third-harmonic generation and, more generally, frequency conversion) have been so far demonstrated only in static or quasi-static configurations, employing slow mechanical or electro-optical modulation schemes. With the exception of a few works,^24−27^ the ultrafast light-by-light reconfiguration of optical nanostructures has been so far limited to the switching of linear functionalities (*e.g.*, light intensity modulation, polarization switching, and so on), mostly employing extended structures including metasurfaces^20,22,28^ (see also refs (29 and 30) for an overview).

## Results and Discussion

**Figure 1:**
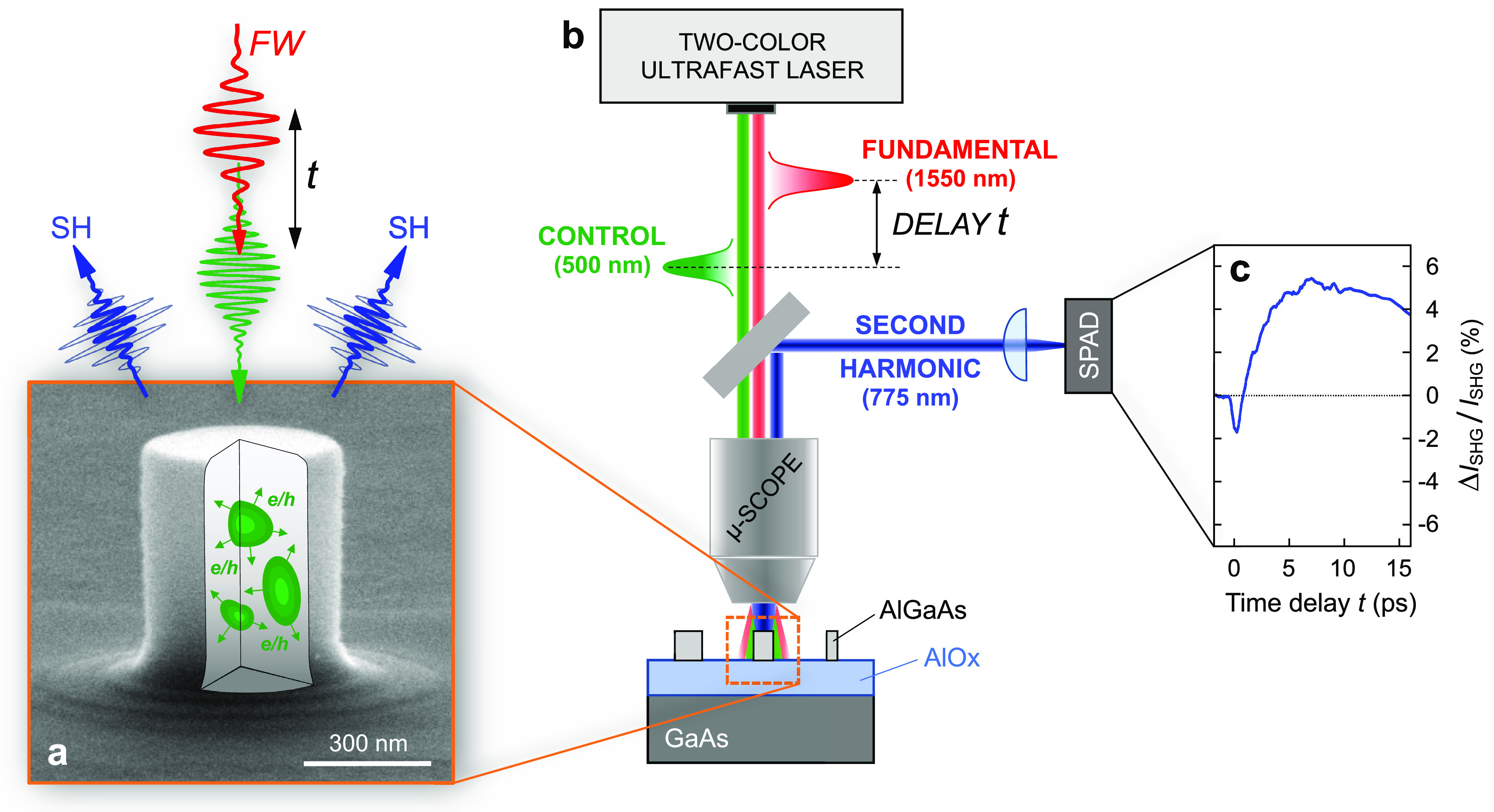
Concept of the ultrafast all-optical control of nanoscale second-harmonic generation. (a) Scanning electron microscopy (SEM) image of the nonlinear nanoantenna made of an AlGaAs nanopillar and sketch of the all-optical control of SHG *via* photoinjection of free carriers (electron–hole pairs) at the nanoscale. (b) Sketch of the experimental setup combining a two-color ultrafast laser source (providing two synchronized femtosecond pulses) coupled to a scanning confocal microscope allowing nonlinear interrogation of selected individual nanoantennas. (c) Sample of the differential SH signal recorded as a function of the time delay between the FW pulse and the control pulse.

Here, we demonstrate efficient and tunable ultrafast all-optical control over SHG at the ultimate limit of nanophotonics, that is, from a single nonlinear nanoantenna. Our concept is illustrated in Figure 1. The nonlinear nanoantenna is a nanopillar made of direct band gap Al_0.18_Ga_0.82_As semiconductor (Figure 1a). The sample consists of an array of well separated (3 μm) structures (therefore behaving as isolated nanoscatterers) with different radii. When irradiated with intense laser light at λ = 1550 nm fundamental wavelength (FW; red pulse in Figure 1a), this configuration has been demonstrated to generate record high SH radiation (λ = 775 nm; blue pulse in Figure 1a) at the nanoscale.^31^ To control SHG from the nanoantenna, we exploit a second ultrashort laser pulse (λ = 500 nm; green pulse in Figure 1a) tuned to a photon energy larger than the band gap of AlGaAs, *E*_G_ = 1.65 eV (corresponding to 751 nm wavelength, for 18% of Al^32^). This pulse is efficiently absorbed *via* interband optical transitions, giving rise to a population of free carriers (electrons in the conduction band and holes in the valence band). These carriers can thus modify the permittivity of AlGaAs at the nanoscale in a broad range of wavelengths, with the most prominent contribution close to the band edge of the semiconductor.^35^ Moreover, thanks to its high refractive index and relatively large size (being in the Mie regime for light scattering), the nanopillar behaves as a multimodal optical nanoantenna. As such, it can provide a more pronounced response to material permittivity changes around its resonances.

To demonstrate all-optical modulation of the ultrafast response of the nanoantenna, we developed a pump–probe setup coupled to a confocal microscope (Figure 1b). The setup is based on a high repetition rate two-branch femtosecond Er:fiber laser,^33^ with one branch delivering 100 fs pulses at the 1550 nm FW and the other branch used to generate synchronized 200 fs pulses at the 500 nm control wavelength. The time delay between the control and the FW pulses is varied by a mechanical delay line, and the two collinear beams are tightly focused (at the diffraction limit) on the plane of the sample by means of a high numerical aperture air objective (see Supporting Information Section S1 for further details). When impinging onto the nanopillar, the FW pulse generates a SH *signal* at 775 nm, which is collected in backward scattering by the focusing objective and subsequently detected with a single-photon avalanche detector (SPAD). From the intensity of the SH light recorded by the SPAD, the relative differential SHG signal is then retrieved as
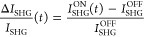
1where *I*_SHG_^ON(OFF)^ is the SHG intensity recorded with (without) control pulse and *t* is the delay between the control and the FW pulses. A typical ultrafast dynamics of the Δ*I*_SHG_/*I*_SHG_ signal is shown in Figure 1c. Further details on the experimental measurement procedure are provided in Supporting Information Section S2.

**Figure 2:**
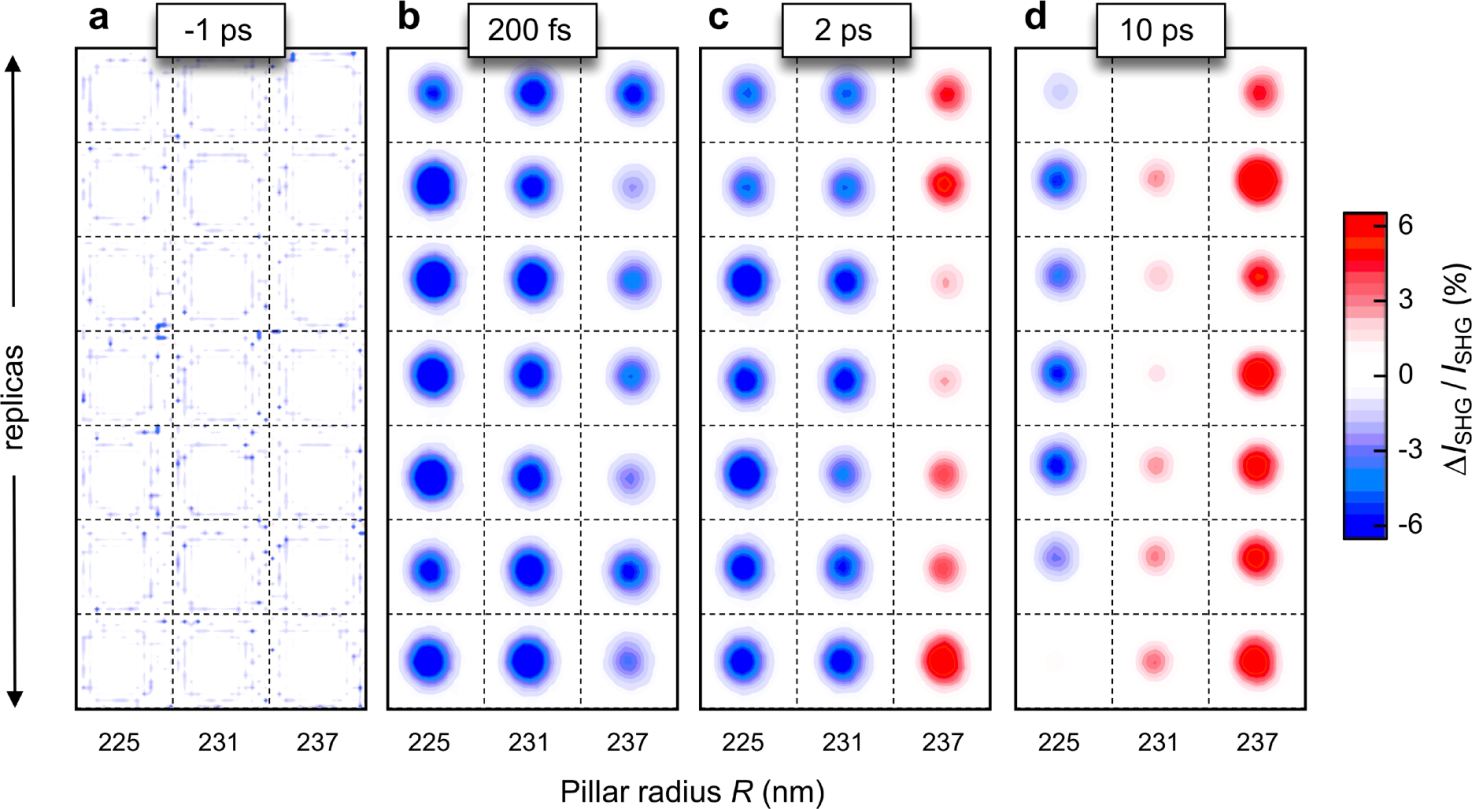
Ultrafast transient SHG in a single AlGaAs nanoantenna. Experimental spatial maps of the ultrafast transient SHG from single AlGaAs nanoanntenas of three different sizes (detailed by pillar radius) recorded for different time delays between control and FW pulses: (a) −1 ps, (b) 200 fs, (c) 2 ps, and (d) 10 ps. For better visualization, we show a grid of cuts (∼1 μm × 1 μm) centered at the individual nanoantennas. Each column in the grid presents different replicas of pillars with the same nominal size.

By scanning the sample in the focal plane, a collection of Δ*I*_SHG_/*I*_SHG_ spatial maps is recorded for a number of different nanopillars and for different time delays, as shown in Figure 2 for four values of *t*: −1 ps (a), 200 fs (b), 2 ps (c), and 10 ps (d). Our measurements reveal an intense ultrafast modulation of the SHG signal, with peaks as high as ±6%, achieved for a very low fluence of the control pulse *F* ≃ 20 μJ/cm^2^. Most interestingly, a slight change in the pillar radius (*R*) results in a dramatic change of the ultrafast dynamics of the SH signal. As an example, for the pillar with *R* = 225 nm we observe a negative Δ*I*_SHG_/*I*_SHG_, *i.e.*, a decrease of SH emission, over the whole temporal dynamics (compare first columns in Figure 2a–d), whereas for the pillar with *R* = 237 nm an instantaneous decrease in the SH signal is observed, which turns into an increase on the picosecond time scale (compare third columns in Figure 2a–d). The pillar with radius *R* = 231 nm exhibits a transient behavior which is intermediate between the two (see second columns in Figure 2a–d). Despite such a dramatic dependence of the transient nonlinear optical response on the pillar size, the reproducibility of our results is ascertained by the fact that nanopillar replicas of the same nominal radius exhibit very similar responses (compare rows in the maps of Figure 2), with the same sign of transient SHG and signal intensity fluctuations < 2% (see Supporting Information Section S2 for further details).

**Figure 3:**
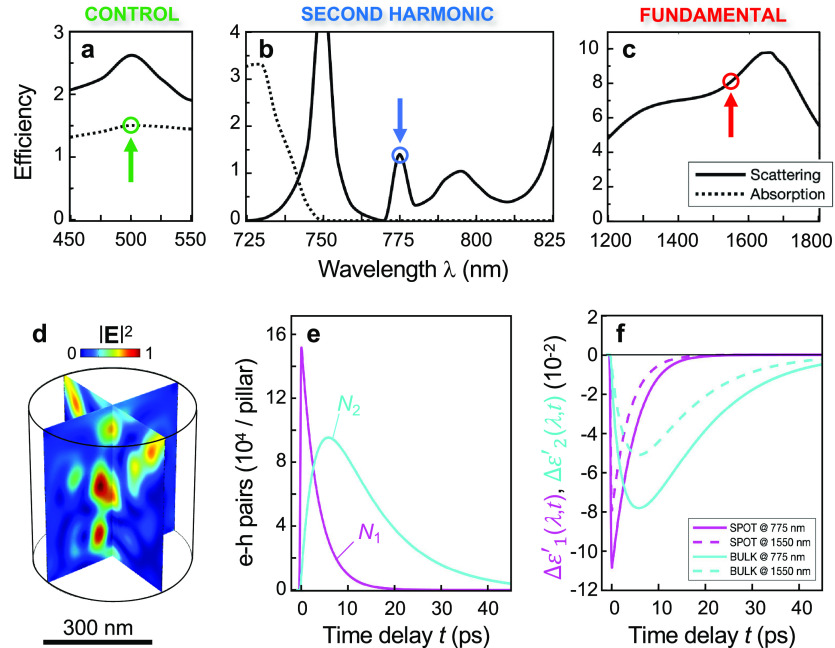
Theoretical model of the all-optical control of SHG. Numerically calculated scattering (solid) and absorption (dotted) efficiencies of the AlGaAs nanoantenna in the spectral ranges of (a) the control pulse, (b) the SH signal, and (c) the FW. The wavelengths of the three optical fields considered in the experiments are marked by arrows. Plane wave excitation is assumed for the spectra in panels a and c, whereas local dipolar excitation is used to retrieve the spectra of panel b. (d) Numerically simulated intensity pattern of the control pulse at 500 nm (to which the absorption pattern is proportional). (e) Temporal dynamics of the electron–hole pairs photogenerated within the hot spots of the control pulse at 500 nm (shown in panel d) *N*_1_ (magenta), and diffused in the whole volume of the nanoantenna, *N*_2_ (cyan). (f) Photoinduced real part permittivity changes arising from *N*_1_, generated in the hot spots (SPOT, magenta) and *N*_2_, generated in the bulk of the nanoantenna (BULK, cyan) evolving over time, evaluated at λ = 775 nm, corresponding to the SH (solid curves) and at λ = 1550 nm, corresponding to the FW (dashed curves). All data are relative to nanoantenna with *R* = 245 nm.

In order to explain such a complex scenario, we developed an *ab initio* model of the SH pump–probe experiments. We considered slightly larger pillar radii compared to the nominal values of the fabricated samples so as to take into account the typical 10–15 nm size increase introduced by our fabrication method.^31^ The simulated absorption and scattering efficiencies for a nanopillar representative of the sizes implemented in our sample (simulated with *R* = 245 nm), evaluated over a suitable wavelength range around the control, signal, and fundamental wavelengths, are shown in Figure 3a–c, respectively (see Supporting Information Section S3 for details). Note in particular a pronounced scattering resonance peaked at 775 nm (Figure 3b), which is dominated by electric quadrupolar Mie response similarly to what is reported by some of the present authors on AlGaAs-on-AlOx nanopillar antennas of comparable sizes.^34^ On the contrary, around the 500 nm wavelength chosen for the control pulse, the nanoantenna behaves as an efficient absorber, with a relatively broad spectral response (Figure 3a). The control pulse at 500 nm thus promotes electrons from the valence to the conduction band of AlGaAs, and a population of electron–hole pairs, *N*_1_, is created in the nanopillar with the spatial distribution of the absorption pattern of the control beam (Figure 3d), which exhibits electromagnetic hot spots where radiation absorption is more efficient. This population evolves over a very short time scale *via* ambipolar diffusion, giving rise to a second population *N*_2_ of hot carriers almost uniformly distributed in the nanopillar. This second population then decays by nonradiative recombination, thus increasing the temperature (*T*) of the AlGaAs lattice.^10,21^ The dynamics of *N*_1_, *N*_2_, and *T* were retrieved by solving a reduced rate equation model, as detailed in Methods (see also Supporting Information Section S4 for details).

With the dynamics of *N*_1_ and *N*_2_ at hand (Figure 3e), we then computed the corresponding modulation of material permittivity (the contribution arising from lattice temperature *T* has instead been neglected since it is quantitatively negligible when compared to the first two, on the time scale of interest). We considered both interband and intraband contributions. The former gives rise to the so-called *band-filling* (Pauli blocking) mechanism due to interband absorption of the control pulse and subsequent variation of occupation probability in the band structure of the semiconductor. The latter effect is modeled in terms of a transient *Drude plasma* formation both in the valence band (hole plasma) and in the conduction band (electron plasma). The permittivity changes arising from the two mechanisms are detailed in Methods.

Results of the calculated complex permittivity changes at 775 nm (solid curves) and at 1550 nm (dashed curves) arising from both *N*_1_ (magenta traces) and *N*_2_ (cyan traces) are shown in Figure 3f. Our model reveals that the relative permittivity modulation is dominated by a real contribution of negative sign from *N*_1_ (magenta traces) and *N*_2_ (cyan traces) for both wavelengths, with peak values of −10.8 × 10^–2^ and –7.8 × 10^–2^ for an incident fluence of 20 μJ/cm^2^, respectively achieved at around 200 fs and 6 ps time delays at 775 nm. In this wavelength range, the permittivity modulation is due to band-filling effect, with negligible contribution from the Drude mechanism, whereas at 1550 nm the band-filling provides only a minor correction to the dominant permittivity change caused by the Drude effect. Regarding the latter, an imaginary permittivity modulation at 1550 nm is also retrieved, but with peak value as low as 6 × 10^–4^, and as such, it has been disregarded. For the band-filling effect, no imaginary permittivity variation is retrieved either at 775 nm or at 1550 nm, since these wavelengths fall in the band gap of the semiconductor.

As a final step of our model, we simulated the variation of the SHG signal caused by the photoinduced permittivity modulations. A perturbative finite-element method numerical analysis was performed on a 3D model of the pillar, assuming the AlGaAs susceptibility tensor χ^2^ from the literature, following the analysis reported in ref (31) (see Methods and Supporting Information Section S5 for further details).

**Figure 4:**
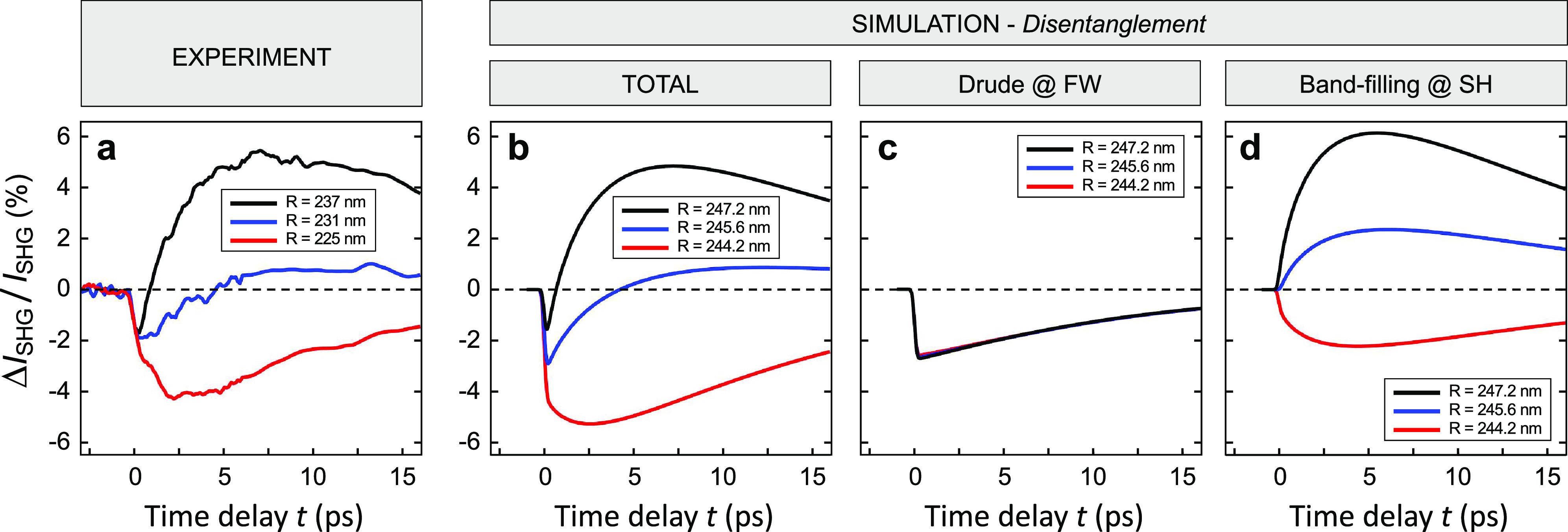
Ultrafast all-optical control of SHG at the nanoscale. (a) Experimental relative differential SHG signal for the three different nanopillars of Figure 2 as a function of the time delay between the control and FW pulse. (b) Simulated relative differential SHG for three different sizes (representative of the three different pillars considered in the experiments), with disentanglement of the two dominant contributions to the all-optical modulation mechanism: (c) Drude plasma formation at 1550 nm and (d) band-filling effect at 775 nm.

Results of the simulations are then compared with the measured dynamics of the transient SHG signal extracted from the data of Figure 2, also comprising several other maps acquired for different time delays (not shown in Figure 2). The experimental Δ*I*_SHG_/*I*_SHG_ dynamics recorded for the three pillars considered in our study (Figure 4a) is indeed well reproduced by our model (Figure 4b), also in terms of the ultrafast sign change observed when moving from the smallest radius (red trace) to the largest one (black trace). Also, the model is capable of reproducing the few picoseconds time delay observed for the onset of the signal peak (in modulus), with respect to the relatively short duration (∼200 fs) of the control pulse. We ascribe this buildup time to the diffusion of electron–hole pairs within the nanopillar and subsequent interplay of effects arising from two populations of carriers, namely, *N*_1_, which is directly coupled to the control pulse and thus localized in hot spots, and *N*_2_, arising from the diffusion of *N*_1_ to the bulk of the pillar. Finally, a disentanglement of the mechanisms presiding over the modulation of AlGaAs dielectric function indicates that the Drude permittivity variation induced at 1550 nm is ultimately responsible for the instantaneous decrease of the SH signal observed for all of the nanoantennas upon absorption of the control pulse (Figure 4c). On the contrary, the band-filling permittivity change induced at 775 nm plays a major role on the SHG modulation for the longer time scale of a few picoseconds (Figure 4d).

This analysis confirms the idea guiding the design of our experiment, according to which efficient all-optical reconfiguration of AlGaAs nonlinear nanoantennas pumped at 1550 nm telecommunication (telecom) wavelength can be achieved *via* photoinjection of free carriers across the semiconducting gap and subsequent giant permittivity modulation at the band edge of the semiconductor, to which the SH wavelength is tuned.

## Conclusions

In summary, we have demonstrated the possibility to reconfigure by all-optical means and at ultrahigh speed the SHG in a single nonlinear nanoantenna. By matching the SH wavelength with the band edge of AlGaAs nanopillars having multiple Mie resonances, a control pulse in the visible is capable of effectively enhancing or quenching (under low control fluence of ∼20 μJ/cm^2^) the SHG efficiency *via* photoinduced permittivity changes. The latter are due to a complex interplay between band-filling effects at the SH wavelength and Drude plasma formation at the FW. The dynamics of the modulated SHG signal is dominated by ambipolar diffusion of the photogenerated electron–hole pairs, leading to the buildup of the transient SH signal within a few picoseconds. The decay of the transient SH signal is governed by nonradiative recombination of the carriers, enhanced by surface states thanks to the higher surface-to-volume ratio at the nanoscale, and thus taking place in a few tens of picoseconds. Also, the sign of the ultrafast modulation of the SHG signal on the picosecond time scale can be controlled by geometrical means, by tuning the nanopillar radius.

Our results can lead to the development of a class of nanophotonic devices whose nonlinear optical response is reconfigured at 10–100 GHz speed, of interest for telecom and sensing applications. First, ultrafast frequency conversion is a key functionality in modern wavelength-division multiplexing for the all-optical transfer of high data rate signals from one frequency carrier to another (see, *e.g.*, ref (36) for an overview). A flat-optics, all optically reconfigurable frequency converter could provide on-chip and even on-fiber integration of optical functionalities. Second, all-dielectric solid-state nanomaterials have been demonstrated to be particularly suitable for high-harmonic generation (HHG) and high-field processes thanks to their high damage threshold.^37^ The kind of control over nonlinear processes at the nanoscale here reported for SHG can thus be extended to HHG as well. Finally, by following the same approach pursued in this work, we envisage ultrafast all-optical control of other second-order nonlinear processes, including difference-frequency generation at the nanoscale and thus the corresponding quantum process of spontaneous parametric down conversion. This could lead to all optically controlled nanosources of entangled photons to be exploited in time-resolved quantum sensing applications.^38^

## Methods

### Fabrication of the SH Nanoantenna

The sample consists of a collection of well isolated (∼3 μm interdistance) Al_0.18_Ga_0.82_As nanopillars with 400 nm height and radius *R* in the (nominal) range 220–240 nm (Figure 1). The nanopillars are supported on a nonstoichiometric aluminum oxide (AlO_*x*_) layer of ∼1 μm thickness and are capped with a thin layer of hydrogen silsesquioxane (HSQ) resist. At the base of the pillar, an AlO_*x*_-AlGaAs interlayer guarantees high mechanical stability (more details on sample fabrication are provided in ref (34)).

### Nonlinear Numerical Model

The nonlinear numerical model is based on a *three*-steps algorithm, for the calculation of (i) the dynamics of the electron–hole pairs photogenerated in the hot spots of the pillar by interband absorption of the control pulse at 500 nm and subsequent diffusion and recombination; (ii) the dynamical permittivity modulation at the SH wavelength induced by the photogenerated electron–hole pairs; and (iii) the intensity of the SHG signal from the 1550 nm pump pulse, with and without the permittivity changes induced by the control pulse.

Step i is based on a reduced model which solves the following rate equations:
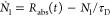
2
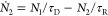
3
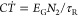
4where the dot denotes time derivative and *T* is the lattice temperature of the nanopillar, with heat capacity *C* = *c*_L_*V*, *V* being the nanopillar volume and *c*_L_ = 1.86 × 10^6^ J/(m^3^ K) the lattice specific heat of AlGaAs.^32^ In the above equations, τ_D_ = 3.6 ps is the effective diffusion time of population *N*_1_, whose order of magnitude has been estimated on the basis of a simple 1D ambipolar diffusion model (see Supporting Information, Section S4), and τ_R_ = 11 ps is the electron–hole nonradiative recombination time, fitted on the experimental data. The source term is the instantaneous rate of photon absorption *R*_abs_(*t*), driving the photoexcited electron–hole population *N*_1_, given by the equation
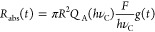
5where *Q*_A_(*hν*_C_) ≃ 1.4 is the absorption efficiency of the nanopillar evaluated at the photon energy of the control pulse *hν*_C_ (λ_C_ = 500 nm), *F* is the fluence of the control pulse, and *g*(*t*) is its temporal normalized intensity profile, reading as
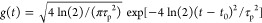
with pulse duration τ_p_ (full width at half-maximum intensity). In the simulations we assumed τ_p_ = 250 fs to take into account the cross-correlation between FW and control pulses.

Step ii is accomplished by resorting to semiclassical modeling of optical transitions in the solid state. Regarding interband transition effects, we have followed the band-filling model detailed in ref (35), under parabolic band approximation with 2-fold contribution, from light holes (LH) and heavy holes (HH), and effective masses taken from ref (32), reading as *m*_e_ = 0.084*m*_0_, *m*_lh_ = 0.099*m*_0_, and *m*_hh_ = 0.573*m*_0_, *m*_0_ being the free electron mass.

First of all, the modulation of the absorption coefficient α arising (for optical frequency ν > ν_G_ = *E*_G_/*h*) from the two photogenerated populations (*N*_*j*_, with *j* = 1, 2) is retrieved as Δα_*j*_ = Δα_LH,*j*_ + Δα_HH,*j*_, with
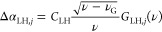
6
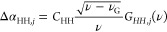
7where
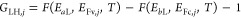
8
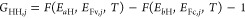
9In the above equations, *C*_LH,HH_ are constants (comprising material parameters, dipole moment matrix element, and fundamental constants) fitted on AlGaAs permittivity data: *C*_LH_ = 3.85 × 10^13^ m^–1^ s^–1/2^; *C*_HH_ = 7.81 × 10^13^ m^–1^ s^–1/2^. *F*(*E*,*E*_F_,*T*) = [1 + *e*^(*E*–*E*_F_) /(*k*_B_ *T*)^]^−1^ is the Fermi–Dirac function at temperature *T* to be evaluated for *E* = *E*_*a*L_, *E*_*b*L_, *E*_*a*H_, *E*_*b*H_, whose expressions, as a function of the optical frequency ν, are given by eqs 6a–b in ref (35). Regarding the quasi-Fermi levels, the lowest order of approximation is assumed, reading as
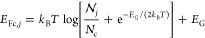
10
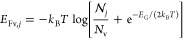
11where  is the average density of the two plasmas arising from the two populations of electron–hole pairs, *N*_1_ and *N*_2_, and  is the effective density of states in the conduction/valence band, defined as in ref (35). From Δα_*j*_, the modulation of the imaginary part of the material’s refractive index is computed as Δ*n*_*j*_^″^ = *c*Δα_*j*_/(4*πν*), *c* being the speed of light in vacuum, and the corresponding modulation of the real part, Δ*n*_*j*_^′^, is then retrieved by Kramers–Kronig analysis. Finally, the permittivity modulation arising from band-filling effect is computed as Δε_B,*j*_ = 2[*n*′Δ*n*_*j*_^′^ – *n*″Δ*n*_*j*_^″^] + i2 [*n*′Δ*n*_*j*_^″^ + *n*″Δ*n*_*j*_^′^].

The photogenerated electron–hole pairs also result in a transient joint plasma of free carriers with density . The corresponding complex permittivity modulation, Δε_D,*j*_ = Δε_D,*j*_^′^ + i Δε_D,*j*_^″^, is thus retrieved by Drude model:^35,39^
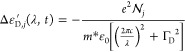
12
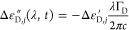
13where m* = (1/*m*_e_ + 1/*m*_h_^*^)^−1^ is the reduced mass of the electron–hole plasma, with *m*_h_^*^ the hole effective mass in the valence band, given by
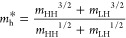
14In eq 12, *e* is the electron charge, ε_0_ is the vacuum permittivity, and Γ_D_ is the Drude damping. The latter is estimated from carrier mobility μ = 2340 cm^2^/(V s)^40^ as Γ_D_ = 1/τ_d_, with τ_d_ = μm*/*e* = 75 fs.

The total permittivity modulations arising from the two populations of carriers, respectively Δε_1_ and Δε_2_, evaluated at the two wavelengths under consideration, *i.e.*, λ = 775 nm (the SH) and λ = 1550 nm (the FW), as a function of the time delay are then computed as
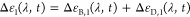
15
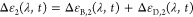
16

The final step iii of our algorithm is based on a 3D finite element method (FEM) numerical analysis in the frequency domain, employing a commercial tool (COMSOL Multiphysics 5.4). At first, the near fields at the FW of 1550 nm are calculated using scattered-field formalism with perfectly matched layer boundary conditions under plane wave illumination, with a linearly polarized electric field parallel to the *x*-axis. These fields are then employed as local source terms by proper definition of the driving nonlinear polarizability using the χ^(2)^ bulk tensor of AlGaAs available from literature, and the fields at the SH wavelength are then calculated. Note that, in view of the zinc blend crystal structure of AlGaAs, the only nonvanishing terms of the χ_*i*,*j*,*k*_^(2)^ tensor are those with *i* ≠ *j* ≠ *k*, whose value in the calculations is set to 100 pm/V.^41^ The crystal axes are oriented as the simulation axes. The SH scattered intensity is then integrated over the solid angle corresponding to the aperture of the microscope objective employed in our experiments. The calculations are repeated after applying a fixed variation in the real part of material permittivity (equal to 0.1) in the hot spots generated by the control pulse (see Figure 3d) or in the whole volume of the pillar (of radius *R*), in order to evaluate the following coefficients:
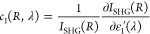
17
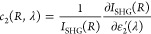
18

It should be noted that these coefficients (detailed in Section S5 and Figure S8) are retrieved for perfect cylindrical symmetry of the pillars and thus represent only an estimation of the transient nonlinear response of the fabricated samples. For example, deviations between experiments and simulations are introduced by non-idealities in the pillar geometry, especially when considering experiments performed on individual nanostructures rather than ensembles or metasurfaces, where the large area excitation results into averaging and compensation of the effects. For this reason, in our calculations the nonlinear coefficients have been weighted by two sets of fitting parameters, *w*_1_(λ), for the hot spots coefficients, and *w*_2_(λ), for the bulk coefficients. We found good agreement between experiments and numerical simulations with the following weighting parameters: *w*_1_(775nm) = 0.2, *w*_1_(1550nm) = *w*_2_(775nm) = *w*_2_(1550nm) = 1. The *w*_1_ coefficient at 775 nm is the most critical and possibly was overestimated in our simulations because of two main reasons. First of all, in view of the higher quality factors of Mie resonances around the SH wavelength, the near-field pattern of the SH signal is expected to be more sensitive to surface roughness and to slight variations of the pillar geometry compared to the pattern of the FW (compare Figure 3b,c). Second, surface scattering losses and residual absorption near the band edge of AlGaAs, not taken into account in our model, can degrade the resonant response at the SH. These issues are in line with the fact that the three different radii considered in our simulations are spread on a much shorter interval of values (from 244.2 to 247.2 nm) compared to the experiments (nominal radii from 225 to 237 nm).

The transient SHG signal is finally computed by multiplying the coefficients from eqs 17 and 18 with the corresponding permittivity modulations of Figure 3f and summing up all four resulting contributions:
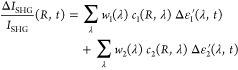
19where λ = 775, 1550 nm.

## Supporting Information Available

Sections S1–S5: Second-harmonic pump–probe setup; transient SH measurements; optical absorption and linear scattering modeling; ambipolar diffusion of electron–hole pairs; modeling SHG modulation (PDF)
