# Supplementary material for: Ultrafast, All Optically Reconfigurable, Nonlinear Nanoantenna
Source: ACS Nano. 2021 Jul 7;15(7):11150–7. doi: 10.1021/acsnano.1c03386 (PMC8397406; doi:10.1021/acsnano.1c03386)
Supplement: Supplementary file 1 — nn1c03386_si_001.pdf [file nn1c03386_si_001.pdf]

Supplementary Information for: Ultrafast, All Optically Reconfigurable, Nonlinear Nanoantenna

Eva Arianna Aurelia Pogna[⋆,†,‡] Michele Celebrano[⋆,†] Andrea Mazzanti[⋆,†] Lavinia Ghirardini,[†] Luca Carletti,[¶] Giuseppe Marino,[§] Andrea Schirato,[†,∥] Daniele Viola,[†] Paolo Laporta,[†,⊥] Costantino De Angelis,[¶] Giuseppe Leo,[§] Giulio Cerullo,[∗,†,⊥] Marco Finazzi,[∗,†] and Giuseppe Della Valle[∗,†,⊥]

†*Dipartimento di Fisica - Politecnico di Milano, Piazza Leonardo da Vinci, 32, I-20133 Milano, Italy*

‡*NEST, CNR-Istituto Nanoscienze and Scuola Normale Superiore, Piazza San Silvestro 12, 56127 Pisa, Italy*

¶*Dipartimento di Ingegneria dell'Informazione, Università di Brescia, Via Branze 38, I-25123 Brescia, Italy*

§*Matériaux et Phénomènes Quantiques, Université de Paris & CNRS, F-75013 Paris, France*

∥*Istituto Italiano di Tecnologia, Via Morego 30, I-16163, Genova, Italy*

⊥*Istituto di Fotonica e Nanotecnologie - Consiglio Nazionale delle Ricerche, Piazza Leonardo da Vinci, 32, I-20133 Milano, Italy*

E-mail: giulio.cerullo@polimi.it; marco.finazzi@polimi.it; giuseppe.dellavalle@polimi.it

S1. Second-harmonic pump-probe setup

The experimental setup for time-resolved SHG is based on a dual branch Er:fiber laser system (Toptica-Femtofiber pro) with each branch generating 300 mW, 100-fs pulses at 1550 nm and 40 MHz repetition rate (Fig. S1). The first branch generates the FW pulses used in the SHG experiment. The second branch is coupled to a highly nonlinear fiber to generate a broadband supercontinuum (SC) covering the 900-1450 nm wavelength range. The SC is sent to a bandpass filter and to a beta-barium borate crystal, for SHG, generating 500-nm light, used as control pulses.

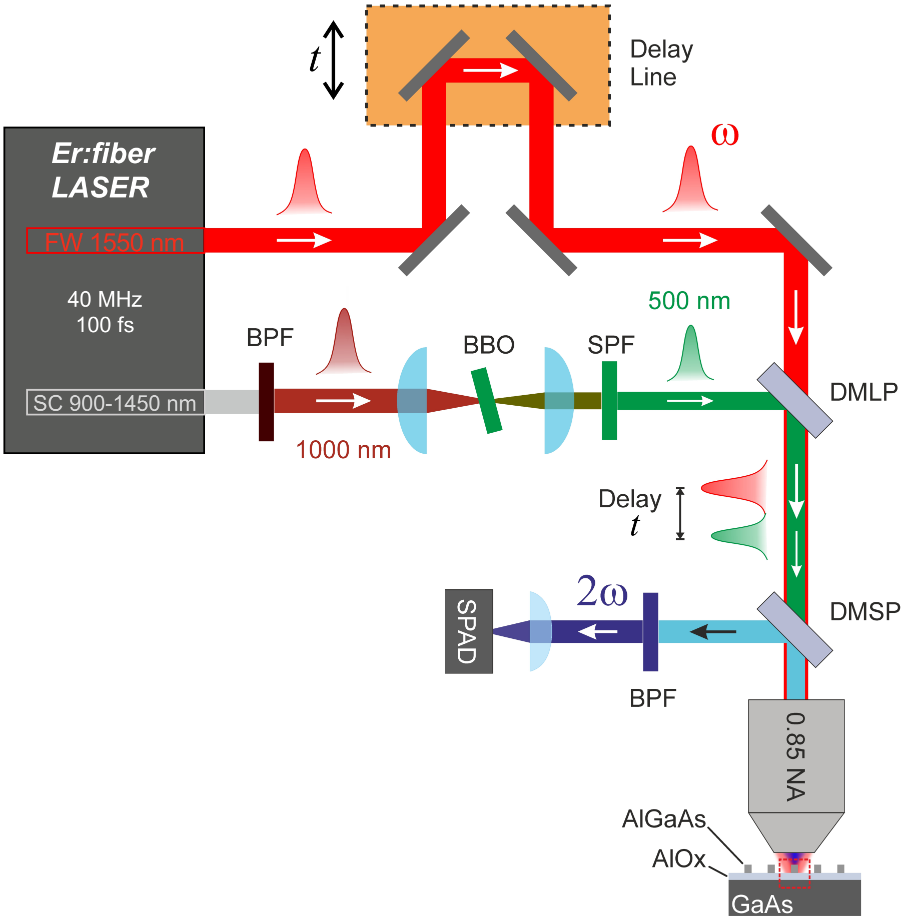

Figure S1: **SH pump-probe experimental setup**. BPF: band pass filter. SPF: short pass filter. BBO: Beta-barium borate crystal. DMLP/DMSP: Dichroic mirror long/short pass. SPAD: Single photon avalanche detector. NA: numerical aperture.

The FW and control pulses are synchronized by a motorized delay stage, collinearly

combined by a dichroic mirror (DMLP 950, Thorlabs Inc.) and focused on the sample by a microscope objective with 0.85 numerical aperture (Olympus UAPON40XO340). The back-scattered SH light is collected by a second dichroic mirror (DMSP 900, Thorlabs) to filter it from the reflected FW beam, and detected, after suitable spectral filtering (775 nm, 25 nm bandwidth, Omega Filters), by a single-photon avalanche diode (SPAD, Micro Photon Devices) coupled to a photon-counting electronics.

S2. Transient SH measurements

The experimental sample consists of several nanopillars organized in columns of replicas sharing the same nominal radius and separated by 3 μm in order to allow for characterizing each of them individually.

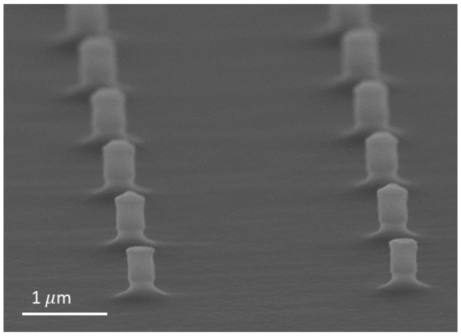

Figure S2: **AlGaAs nanoantenna sample.** SEM image of the sample showing the array arrangement of individual nanopillars, separated by 3 μm distance.

Figure S2 reports images of the sample acquired by scanning electron microscopy (SEM) and Fig. S3 shows a map of the intensity of the SHG signal acquired with the confocal microscope illuminating the sample with 1550 nm light and collecting the generated SH at 775 nm. This map demonstrates the setup capability to collect the emission from individual structures. From the I_{SHG} maps we could extract, by scanning the edge of one nanopillar, line profiles as the one reported in Fig. S4.

We analysed the line profile using a rectangular function with edge equal to the pillar diameter convoluted with a Gaussian function describing the beam spot with full-width half maximum (FWHM) equal to $\Delta = 687 \pm 1$ nm, which can be assumed as an estimation of the spatial resolution of the setup.

The variation of the transient SHG signal within seven nanopillar replicas with identical nominal radius is analysed as a function of the time delay. In Fig. S5, we report the transient signal of the three sets of nanopillars evaluated by averaging the maps of Fig. 2 in a 400 nm circular area around each replica, for the three different time delays ($t = 200$ fs, 2 ps, 10 ps). The mean values of transient SHG signal for the three radii and the three time delays is

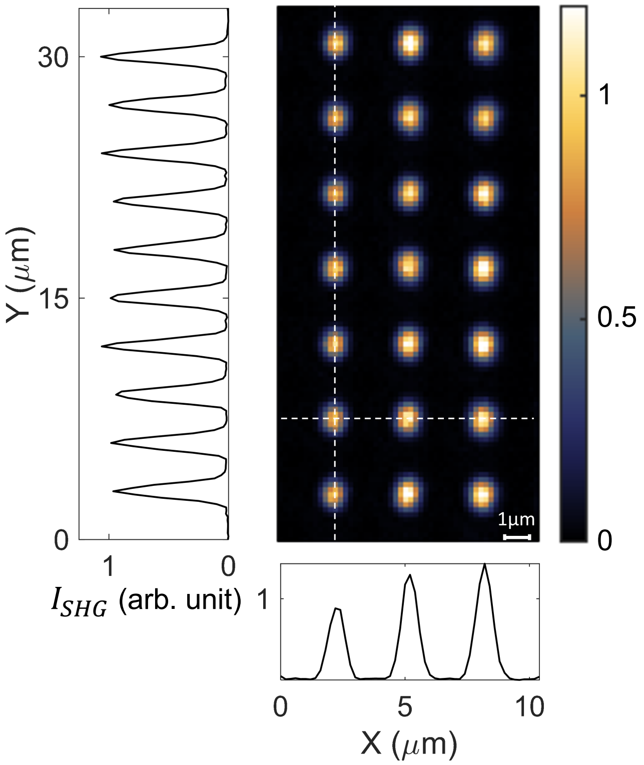

Figure S3: **AlGaAs nanoantennas.** Map of the measured SHG, including 7 replicas of 3 sets of nanopillars with radius $R = 225, 231, 237$ nm increasing from left to right; together with the line profiles extracted cutting the map along the white dashed lines vertically (left panel) and horizontally (bottom panel).

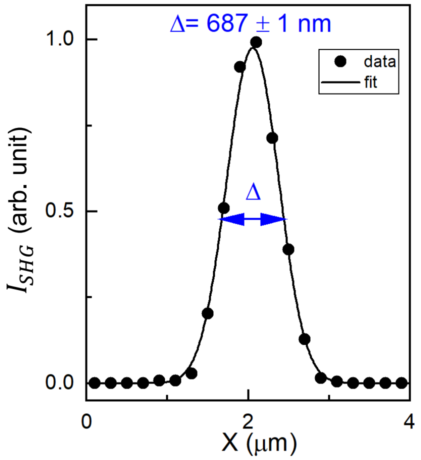

Figure S4: **Spatial resolution of transient SHG microscopy measurement.** Linecut of the SHG intensity map in Fig. 2 intersecting a pillar of radius 225 nm (black dots and line), analysed with the fit-function described in the text to estimate the spatial resolution $\Delta = 687 \pm 1$ nm of our SHG microscopy measurement.

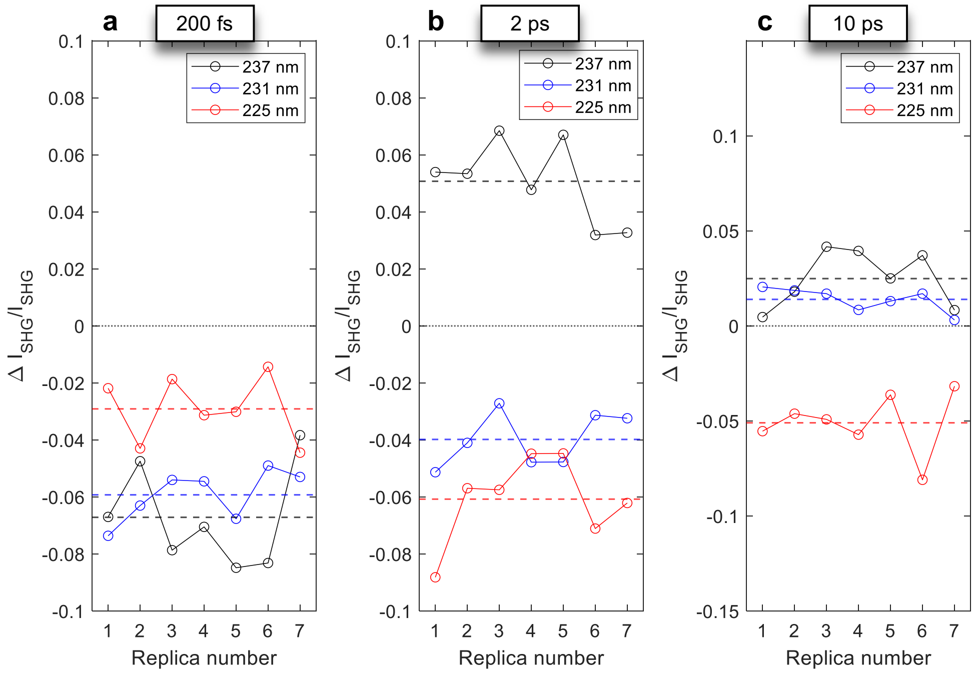

Figure S5: **Ultrafast transient SHG signal fluctuations** at three different delays (a) t = 200 fs, (b) t = 2 ps and (c) t = 10 ps, for nanopillar replicas with nominal radius: (red dots and solid lines) r = 225 nm, (blue dots and solid lines) r = 231 nm and (black dots and solid lines) r = 237 nm extracted from the maps of Fig. 2. The average signal for each set of nanopillars and time delay is shown as dashed colored line.

reported in Table 1 together with the standard deviation, which is $<2\%$.

Table 1: Ultrafast transient SHG signal average and standard deviation evaluated on a set of seven nanopillar replicas for each nominal radius: $r = 225$, 231, 237 nm and time delay $t = 200$ fs, 2 ps, 10 ps.

$\Delta I_{SHG}/I_{SHG}$	**225 nm**	**231 nm**	**237 nm**
**200 fs**	-0.029±0.012	-0.059±0.009	-0.067±0.018
**2 ps**	-0.061±0.015	-0.040±0.009	0.051±0.015
**10 ps**	-0.051±0.016	0.014±0.006	0.025±0.015

The time-dependent SHG intensity $I_{\mathrm{SHG}}(t)$ after photoexcitation is estimated by acquiring, at each time-delay, an image of the SH generated by the single pillar scanning an area of 1×1 μm^2 with 8×8 pixels around it, in order to compensate for misalignment induced by mechanical vibrations. Each pixel was integrated for 60 ms for a total scanning time of 5 s for each map. Three examples of $I^{ON}_{\mathrm{SHG}}(t)$ maps of a nanopillar with 225 nm radius,

acquired at three time delays, are reported in Figs. S6a-c. From each map, we retrieved the average intensity calculated on the pixels with values higher than the 10% of the maximum, to reduce the contribution from background. We finally obtained the relaxation dynamics of the transient $\Delta I_{\mathrm{SHG}}(t)$ by calculating the difference between the value $I^{ON}_{\mathrm{SHG}}(t)$ extracted at time delay t and the intensity I^{OFF}_{SHG} extracted from the map acquired when blocking the pump, see Figs. S6d-e.

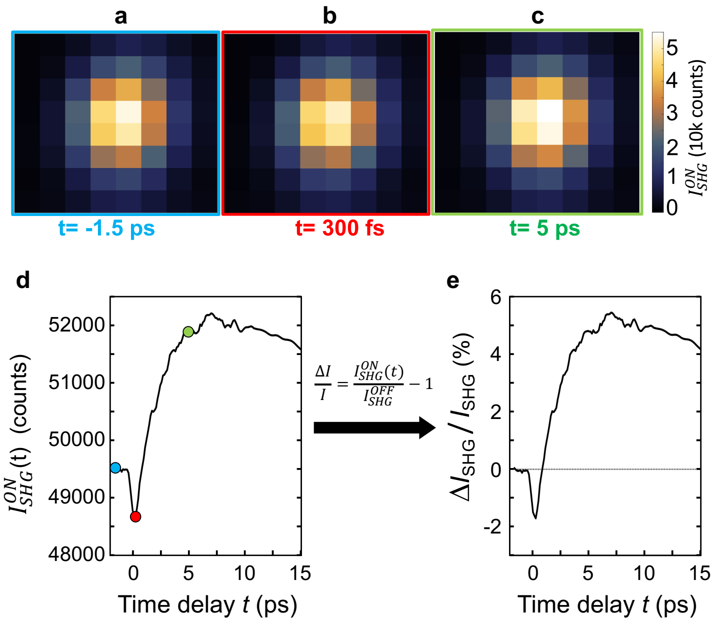

Figure S6: **Determination of SH dynamics. a-c,** Three maps of the SH intensity emitted by a nanopillar of radius 225 nm at three different time delays with respect to the pump-pulse: $t = -1.5$ ps (a), $t = 300$ fs (b), $t = 5$ ps (c). **d,** Transient SH signal dynamics $I^{ON}_{SHG}(t)$ (black-line), calculated by taking the weighted average of I^{ON}_{SHG} maps recorded at different time delays, as those in panels (a)-(c) (solid circles). **e,** Relative transient SH signal obtained from $I^{ON}_{SHG}(t)$ by subtracting the intensity measured without the pump and normalizing the difference for it.

S3. Optical absorption and linear scattering modeling

Optical absorption and linear scattering from AlGaAs nanoantennas are modeled with full-vectorial numerical analysis based on a finite-element-method implemented in a commercial tool (Comsol Multiphysics). The refractive index of AlGaAs is taken from experimental data,[1] while the refractive index of the substrate is set at 1.6 and is assumed constant at all wavelengths. Perfectly matched boundary conditions are used at the physical boundaries of the simulation to represent open boundaries. The optical excitation is modeled as a plane wave with an electric field linearly polarized along the x-axis and k-vector parallel to the nanopillar axis. The scattering and absorption efficiencies are evaluated as:

$$Q_{sca} = \frac{\int_A \vec{\mathbf{S}} \cdot \hat{\mathbf{n}} \mathrm{d}A}{I_0 \pi R^2}, \tag{1}$$

and

$$Q_{abs} = \frac{\frac{1}{2}\int \sigma |\vec{\mathbf{E}}|^2 \mathrm{d}V}{I_0 \pi R^2}, \tag{2}$$

where $\vec{\mathbf{S}}$ is the Poynting vector, A is a closed surface enclosing the resonator whose normal outward direction is $\hat{\mathbf{n}}$, I_0 is the intensity of the incident plane wave, R is the resonator radius, σ is the AlGaAs conductivity, $\vec{\mathbf{E}}$ is the local electric field and V is the nanoresonator volume.

S4. Ambipolar diffusion of electron-hole pairs

To estimate the timescale of electron-hole diffusion in the nanopillar, we developed a reduced model of ambipolar diffusion. Since the hot spots generated by the control pulse are mostly concentrated in the central section of the pillar (Fig. 3d), we assumed that the process is governed by vertical diffusion. Being z the vertical axis of the cylinder, the spatio-temporal evolution of electron-hole concentration $\mathcal{N}$ inside the nanopillar can be described by the

following equation:

$$\frac{\partial \mathcal{N}(z,t)}{\partial t} = G(z,t) - \frac{\mathcal{N}(z,t)}{\tau_R} + D\frac{\partial^2 \mathcal{N}(z,t)}{\partial z^2}, \tag{3}$$

with G the driving term due to the impulsive photoexcitation, that we modeled as:

$$G(z,t) = G_0 \exp\left(-\frac{2(z-z_0)^2}{\delta^2}\right) \exp\left(-\frac{2(t-t_0)^2}{\tau_0^2}\right). \tag{4}$$

In the above equations, $\tau_0 = 210$ fs (corresponding to $\tau_p = 250$ fs FWHM, in agreement with experimental conditions), $t_0 = 3\tau_0$, τ_R is the electron-hole recombination time, and $D = 18$ cm^2/s is the ambipolar diffusion coefficient. Note that for D we assumed the value measured in GaAs at room temperature[2] to estimate the AlGaAs one, given the relatively low concentration of Al in our samples and the more impactful approximation involved in the one-dimensional modeling. In order to mimic the spatial localization of the hot spots in the central section of the pillar, we set $z_0 = 200$ nm (corresponding to the center of the pillar) and $\delta = 160$ nm (corresponding to 40% of the pillar height). Finally, note that, in view of the linearity of Eq. (3) and for the aims of our analysis (that is the estimation of the time scale of electron-hole homogeneisation in the pillar), the peak of electron-hole photogeneration rate per unit volume, G_0, can be set to a unitary value without loss of generality.

Equation (3) is solved with Neumann boundary conditions on the z-axis interval $[0, 400]$ nm, and for $t > 0$, and the time- and space-dependent (normalized) carriers concentration $\mathcal{N}(z,t)$ is retrieved (Fig. S7a).

We defined then two populations of carriers, N_1, comprised in the section δ (integration domain $[\delta]$), and N_2, distributed in the rest of the pillar (integration domain $1-[\delta]$), as:

$$N_1(t) = \pi R^2 \int_{[\delta]} \mathcal{N}(z,t)\mathrm{d}z, \tag{5}$$

$$N_2(t) = \pi R^2 \int_{1-[\delta]} \mathcal{N}(z,t)\mathrm{d}z, \tag{6}$$

where R is the radius the nanopillar (~ 200 nm). The results of this calculation, in the

absence of recombination processes, are detailed in Fig. S7b-c (dotted curves).

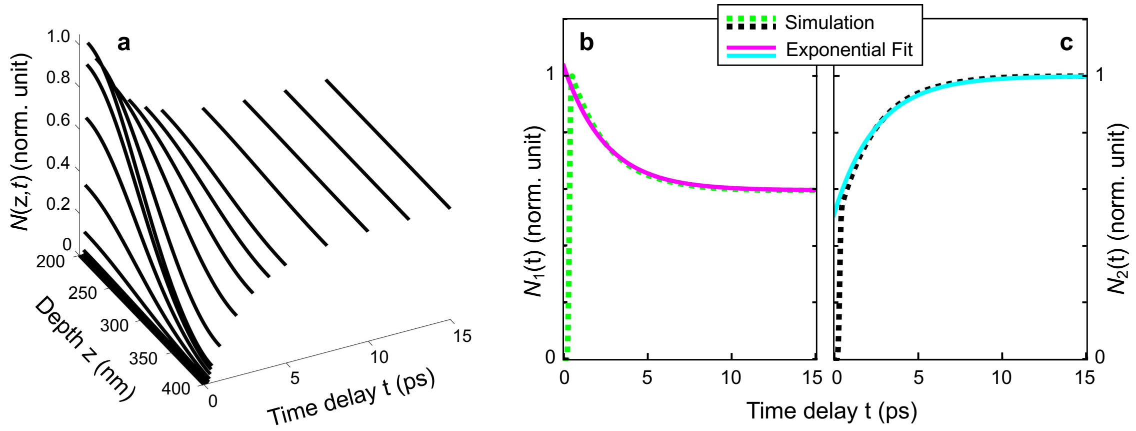

Figure S7: **Model order reduction of ambipolar diffusion.** **a**, Simulated spatio-temporal dynamics of carriers diffusion according to the 1D model of Eqs. (3)-(4), neglecting electron-hole recombination. The spatial profile of carriers density (normalized to the peak value) is shown at selected time delays. **b,c**, Identification of effective diffusion time constant: numerical solutions from Eqs. (5)-(6) (dotted curves) are compared with exponential fits (solid curves) with the same time constant for the two populations.

After the initial transient due to photoexcitation, the dynamics of N_1 and N_2 can be well approximated with exponential curves, having the same time constant τ_D (solid curves in Fig. S7b-c). A fitting procedure for time delays $t > 0.5$ ps retrieves $\tau_D = 2.5$ ps. Note that this simplification, according to which the diffusion process can be reduced to two populations of carriers with temporal dynamics described by one single time constant, is at the basis of the rate equations model of Eqs. (2)-(3) in the main text. Of course, in principle, carrier recombination can occur during diffusion as well. However, in agreement with reference,[3] we assumed trap-assisted non-radiative recombination as the dominant process, with a time constant $\tau_R = 11$ ps estimated on the basis of the decay dynamics observed in the experiments. Therefore, being the recombination time much longer than the diffusion time, a separation of the temporal scales of the two processes is legitimate. Finally, note that our estimation of τ_D is not aimed at providing an accurate value for the diffusion time, but to establish the foundational idea of the rate equations model and to determine the time scale of carriers diffusion. In the simulations reported in the main text we found good agreement

with the experiments when using $\tau_D = 3.6$ ps, which is in line with the fact that diffusion in the pillar takes place also along the radial direction.

S5. Modeling SHG modulation

The coefficients detailed by Eqs. (17)-(18) in the main text represent a numerical estimation of the partial derivative of the SHG intensity I_{SHG} with respect to permittivity variations in the hot spots of the control laser beam or in the bulk of the nanopillar, normalized to the SHG intensity from the unperturbed structure. The results of our calculations are summarized in Fig. S8. When evaluated at the FW (1550 nm, dashed curves in Fig. S8), these coefficients are positive and almost insensitive to both the pillar radius and the spatial configuration of the permittivity change. This is in line with the fact that the pillars exhibit a very shallow resonant behavior in this wavelength range (cf. Fig. 3c). On the contrary, at the SH wavelength of 775 nm, where the pillar resonances are sharper (cf. Fig. 3b), the nonlinear coefficients (solid curves in Fig. S8) turned out to be strongly dependent on pillar size and assume both positive and negative sign depending on R. Note that the spatial pattern of permittivity modulation at 775 nm also plays a role: the coefficient from localized permittivity modulation in the hot spots of the control beam (solid magenta trace) and the coefficient from homogeneously distributed permittivity change (solid cyan trace) cross zero for different values of R.

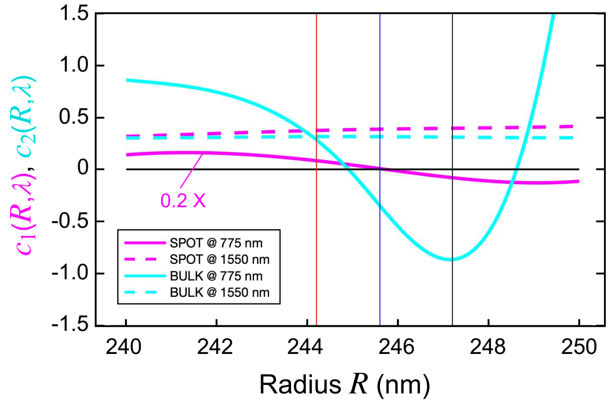

Figure S8: **SH modulation coefficients.** Calculated nonlinear spectral coefficients for SHG modulation following real part permittivity variation in the hot spots (magenta) and in the whole bulk (cyan) at the SH (solid curves) and at the FW (dashed curves). Vertical lines mark the three selected values of the pillar radius R considered in the simulations of Fig. 4 (same color coding as for the traces of Fig. 4).

References

1. Gehrsitz, S.; Reinhart, F. K.; Gourgon, C.; Herres, N.; Vonlanthen, A.; Sigg, H. The Refractive Index of $Al_xGa_{1-x}As$ below the Band Gap: Accurate Determination and Empirical Modeling. *J. Appl. Phys.* **2000** *87*, 7825.

2. Ruzicka, B. A.; Werake, L. K.; Samassekou, H.; Zhao, H. Ambipolar Diffusion of Photoexcited Carriers in Bulk GaAs. *Appl. Phys. Lett.* **2010** *97*, 262119.

3. Shcherbakov, M. R.; Liu, S.; Zubyuk, V. V.; Vaskin, A.; Vabishchevich, P. P.; Keeler, G.; Pertsch, T.; Dolgova, T. V.; Staude, I.; Brener, I.; Fedyanin, A. A. Ultrafast All-Optical Tuning of Direct-Gap Semiconductor Metasurfaces. *Nat. Commun.* **2017** *8*, 17.
